# P09 A catastrophic decline: a significant skin rash in a patient presenting with catastrophic antiphospholipid syndrome

**DOI:** 10.1093/rap/rkad070.030

**Published:** 2023-09-27

**Authors:** Nikita Goel, Ravinder Sandhu

**Affiliations:** Russells Hall Hospital, Dudley, United Kingdom; Russells Hall Hospital, Dudley, United Kingdom

## Abstract

**Introduction:**

Catastrophic antiphospholipid syndrome, otherwise knows as CAPS, is a rapidly progressive systemic condition characterised by ischaemic tissue necrosis and microvascular thrombosis. Due to the rarity of the condition, it is often misdiagnosed, leading to high levels of morbidity and mortality. Awareness of the triggers of CAPS as well as the diagnostic tools and management is key to preventing multiorgan failure and mortality. Due to the paucity of cases worldwide and thus randomised controlled trials, there are limited data on management and long term follow up. This case study highlights a patient with CAPS who was managed successfully by an MDT.

**Case description:**

A 35-year-old Caucasian male smoker presented with headache. He had a past medical history of aortic and left external iliac artery clot, with positive lupus anticoagulant 5 years previously. Warfarin had been commenced and he did not attend rheumatology clinic.

Initial observations were within normal parameters and clinical examination revealed neck stiffness, photophobia and diplopia. He had presented to another hospital a week prior with the same symptoms and CT venogram ruled out venous sinus thrombosis. Given his symptoms, he was initially managed for viral/bacterial meningitis with aciclovir and ceftriaxone. CT head and lumbar puncture didn’t reveal any abnormality. Due to ongoing symptoms he had an MRI/MRA on day 5 of admission showing subdural parafalcine haematoma. His warfarin was stopped and he was given vitamin K and prophylactic heparin.

Following on from this, he quickly developed multi-system symptoms including left pleuritic chest pain and oxygen requirement with negative CTPA. His platelets and haemoglobin started to drop and a new rash with palpable purpura appeared on the buttocks, abdomen and left heel. Cardiology concluded he had suffered a type 2 myocardial infarction. Due to new onset confusion, further MRI revealed a new focal infarct in the left external capsule and medial left occipital lobe. Skin biopsy showed vascular thrombosis with vessel wall damage. Immunology showed ANA +ve 1:320, dsDNA +ve, lupus anticoagulant +ve. A diagnosis of CAPS was made. On the basis of refractory thrombocytopenia, IVIG was administered and high dose steroids were commenced. The short-lived response in platelet count to IVIG was superseded with rituximab, treatment dose heparin and IV steroids. The patient remained stable with no further organ involvement or cytopenias and was discharged. In the outpatient setting, the patient opted to continue treatment dose low molecular weight heparin in lieu of warfarin.

**Discussion:**

Due to the patient being treated elsewhere for his previous clots, the positive lupus anticoagulant was unknown at presentation. When the haematoma was diagnosed on MRI/MRA initially, the stroke team input was to stop the warfarin which was necessary to reduce potentiating the bleed further. At the point of rheumatology involvement, it was clear that there were no overt features of sepsis or infection with a relatively normal procalcitonin. The first difficult decision came in commencing steroids when infection was still the primary diagnosis with elevated inflammatory markers. The basis for steroids was the positive immunology, normal PCT, negative infection screen and the development of multiorgan pathology. Further to this, when the infarcts were diagnosed there was a challenging conversation with haematology, who were not comfortable to increase anticoagulation given anaemia and dropping platelets. Our biggest concern at the time was the development of further infarcts and microvascular thrombosis. The skin involvement developed rapidly and without steroids and anticoagulation; it posed the risk of breaking down the abdominal wall with open wounds and increased infection risk.

At the time of diagnosis we initially selected IVIG given the refractory thrombocytopenia and quickly made a decision to escalate to rituximab once the platelets and inflammatory markers rebounded after treatment. There was a paucity of data and clinical guidelines on CAPS given the low number of cases worldwide, but using UptoDate and expert based cases helped to clarify a treatment plan.

The gold standard anticoagulation for antiphospholipid syndrome is warfarin. However, despite patient education, the patient was unhappy to go back to warfarin and continues on low molecular weight heparin, which poses risk of injection site reactions, osteoporosis and electrolyte abnormality. This raises the question of patient choice and the need for ongoing patient education in light of clear guidelines for management.

**Key learning points:**

Catastrophic antiphospholipid syndrome is a multisystem condition driven by complement activation. The main triggers include cessation of anti-coagulation; as in this case, infection, surgery, trauma, pregnancy, malignancy, and autoimmune disease flare. It may be commonplace for doctors in other specialties to advise holding anticoagulation for routine or elective procedures, without considering the perhaps fatal consequences of doing so for patients with antiphospholipid syndrome.

In regards to the diagnosis of this rare condition, it is important to consider the diagnostic criteria: inclusive of the involvement of 3 or more organs; systems or tissues that develop manifestations within less than a week; positive antiphospholipid antibodies; histopathological confirmation. Skin biopsy can be done quickly to provide histology with substantial diagnostic gain compared to internal organ biopsy which poses bleeding risk. It is also important to consider more common mimcs including thrombotic thrombocytopenic purpura, haemolytic uraemic syndrome, sepsis, cholesterol emboli and disseminated malignancy.

In the absence of evidence-based guidelines for rarer conditions, it is imperative to research expert testimonials, cohort studies and the up to date literature. The currently accepted management is triple therapy inclusive of steroids, anticoagulation which is heparin infusion or low molecular weight heparin in the acute phase, followed by warfarin once stabilised and IVIG or plasmapheresis. Further immune suppression such as rituximab can be used in refractory cases, similar to our patient.

Lastly, this case clearly highlights how the multidisciplinary team came together to manage a complex and unwell patient. The team was led by rheumatology who were the key decision makers, but the diagnosis could not have been sought in isolation.

By attending the conference I hope to gain more knowledge through other interesting cases and further my differential diagnoses and lateral thinking when reviewing complex patients as a rheumatology registrar and beyond.

